# Prevalence of insulin resistance in Chinese solar greenhouse and field workers: evidence from a solar greenhouse and field workers study

**DOI:** 10.3389/fpubh.2023.1257183

**Published:** 2023-08-25

**Authors:** Tenglong Yan, Minghui Wang, Siwen Yang, Yuqian Wang, Xingfan Zhou, Xiaojun Zhu, Wenjun Ma, Shichuan Tang, Jue Li

**Affiliations:** ^1^Beijing Institute of Occupational Disease Prevention and Treatment, Beijing, China; ^2^Department of Neurology, Sanbo Brain Hospital, Capital Medical University, Beijing, China; ^3^National Center for Occupational Safety and Health, National Health Commission of the People’s Republic of China, Beijing, China; ^4^Beijing Key Laboratory of Occupational Safety and Health, Institute of Urban Safety and Environmental Science, Beijing Academy of Science and Technology, Beijing, China; ^5^Department of Occupational and Environmental Health Sciences, School of Public Health, Peking University, Beijing, China

**Keywords:** agriculture, occupational epidemiology, solar greenhouse workers, field workers, triglyceride-glucose index, prediabetes, insulin resistance

## Abstract

Evidence suggests that agricultural workers are at higher risk of insulin resistance (IR), but few studies have investigated IR in solar greenhouse workers, who are exposed to higher concentrations of agricultural risk factors than traditional agricultural workers. A prevalence study was conducted in a greenhouse vegetable farm in China. In total, 948 participants were enrolled in this study. Among them, 721 participants were allocated to the greenhouse worker group (G group), and 227 participants were assigned to the field worker group (F group). The TyG index, which is an indicator to evaluate prediabetes (IR), was calculated by the formula: TyG index = ln [fasting triglycerides (mg/dL) × fasting plasma glucose (mg/dL)/2]. To evaluate the associations of TyG index alternation with solar greenhouse and field work, multiple linear regression (MLR) and logistic regression models were performed. The TyG index in the G group (8.53 ± 0.56) was higher than that in the F group (8.44 ± 0.59) (*p* < 0.05). Solar greenhouse work was positively associated with an increased TyG index in both the multiple linear regression model [*β* = 0.207, (0.006, 0.408)] and the logistic regression model [*OR* = 1.469, (1.070, 2.016)]. IR was associated with the solar greenhouse work. However, the determination of agricultural hazard factors needs to be further strengthened to improve exposure assessment.

**Graphical Abstract:**
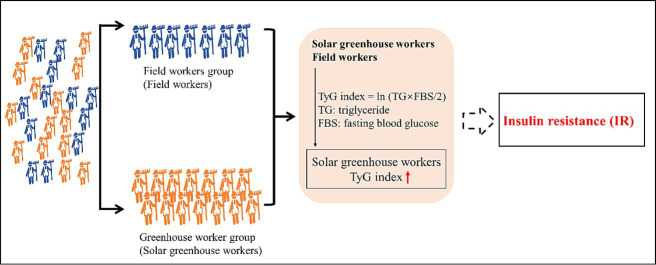

## 1. Introduction

Solar greenhouse cultivation is a technology that originated in the 13th century and has since evolved into a commercial-scale operation. With an estimated 496,800 hectares of greenhouse vegetable production in 2019 (1), 80% of which is located in China, Spain, the Republic of Korea, Japan, Türkiye, Italy, Morocco, and France (2), solar greenhouses enhance productivity and ensure a year-round supply of high-quality agricultural products. However, this technology has not necessarily been accompanied by sound occupational practices, leading to higher levels of pesticide and chemical fertilizer residues than on traditional open-field farms (2). As a result, solar greenhouse workers are exposed to high concentrations of these chemicals over long periods (3), resulting in health issues such as reproductive disorders (4), chronic kidney disease (5), respiratory symptoms (6, 7), neurological symptoms (8, 9), and skin irritation (10).

Insulin resistance (IR) is a state of decreased sensitivity and responsiveness to insulin concentrations (11) that occurs before a person is diagnosed with type 2 diabetes mellitus (T2DM) (12). At least 86 million adults are affected by IR in the U.S. alone, with high rates of undiagnosed cases (13). Increasing evidence suggests that IR is at the crossroads of metabolic syndromes (MetS) (e.g., obesity, diabetes mellitus, hypertension, and cardiovascular disease) in humans, affecting insulin-regulated pathways and many organs once IR is elevated (14–17). The World Health Organization has included IR in its criteria for the diagnosis of MetS (18). However, the IR status of solar greenhouse workers has not been determined, even though the risk factors in solar greenhouses are high.

Exposure to heat and pesticides has been shown to affect insulin sensitivity and the prevalence of T2DM (19, 20) in the general population. Pallubinsky et al. reported that passive moderate heat exposure improved glucose metabolism and insulin sensitivity in overweight humans (20). Exposure to organophosphorus pesticides (OPs) affects the formation of glycation end products, accumulation of lipid metabolites, activation of inflammatory pathways, and oxidative stress, and ultimately causes IR and T2DM (21). The concentration of pesticide chemical residues, temperature, and other risk factors in the solar greenhouse is higher than in the field farm. This study hypothesizes that solar greenhouse workers are likely to be at increased risk of IR compared to field workers due to higher levels of pesticides and chemical residues, temperature, and other risk factors in the solar greenhouse. This study used the triglyceride-glucose (TyG) index, a parameter derived from triglyceride (TG) and fasting blood glucose (FBS) levels that has been proven to be a convincing and reliable indicator of IR (11). The TyG index has been established as a hallmark of T2DM, even several years before the onset of diabetes (22). Hence, this study evaluated whether the prevalence of IR was higher among solar greenhouse workers than among field workers. Both solar greenhouse workers (greenhouse worker group) and field workers (field worker group) living in the same area were recruited for this cross-sectional study. Furthermore, the association between solar greenhouse work and field work with IR was explored. The findings of this study may provide clues to the risk of IR and hypotheses for future prospective epidemiologic research in agricultural communities.

## 2. Methods

### 2.1. Study design and questionnaires

A prevalence study program, known as the Solar Greenhouse and Field Workers Study (SGFW study) (9), was conducted in northwestern China among individuals involved in solar greenhouse vegetable cultivation or field farming. Solar greenhouse and field workers were enrolled in this study, all of whom participated in the routine health check program organized by the local government (5, 6, 9). Solar greenhouse workers and field workers lived in the same area, no more than 5 km apart, and the two groups of workers shared the same dietary habits. The main tasks of the field workers and solar greenhouse workers were to cultivate corn, vegetables, and other crops, with other activities ranging from sowing to harvesting. The workers in the solar greenhouse group worked more than 8 h per day on average, and the workers in the field group also worked more than 8 h per day during busy farming seasons. The study protocol was approved by the Medical Ethics Committee of the National Center for Occupational Safety and Health, National Health Commission of the People’s Republic of China.

This study enrolled a total of 975 participants who met the following inclusion criteria: (i) had lived in the area for at least 1 year; (ii) were 18 years of age or older; (iii) were not pregnant if women; (iv) were engaged in either solar greenhouse work [the solar greenhouse worker group (G group)] or crop production [in the field worker group (F group)]; (v) had undergone blood biochemical testing. Participants who had been diagnosed with diabetes (*n* = 27) were excluded to avoid the potential impact of diabetes treatment on relevant serum biomarkers. Diabetes was defined as self-reported diabetes or FBS ≥ 7.0 mmol/dL according to the diagnostic criteria for diabetes (the Chinese Guidelines for the Prevention and Treatment of Type 2 Diabetes, 2020 Edition). Finally, a total of 948 participants were included in this study, with 721 participants in the G group and 227 in the F group, respectively.

A structured questionnaire was used to collect information on participant characteristics, including demographic characteristics (age, gender, weight, height, educational status), habits (smoking habits, alcohol consumption habits), and pesticide use. Trained interviewers conducted the questionnaire. Body mass index (BMI) was calculated by the formula: BMI = weight (kg)/ height squared (m^2^). The questionnaire included inquiries about 10 types of pesticides commonly used in plantation agriculture, and further details can be found in Supplementary Table S1.

### 2.2. Triglyceride-glucose index

After an overnight fast, 3 mL of blood samples were collected from each participant, and TG and FBS were measured using a Tecom TC6010L automatic analyzer (Tecombio, China) and Tecom diagnostic reagent following standard experiment procedures provided by the manufacturer. The TyG index was calculated as ln [fasting TG (mg/dL) × FBS (mg/dL)/2] (23). Based on the median TyG index, participants were divided into a low TyG index group (TyG index ≤8.49) and a high TyG index group (TyG index >8.49).

### 2.3. Statistical analysis

The data were managed using Epidata 3.1 and analyzed using SPSS 24.0 (SPSS Inc., Chicago, IBM). The Shapiro–Wilk test (S-K test) was used to test the normality of the data. For normally distributed data, the mean ± standard deviation was used to express the results. To test for differences in categorical parameters, the chi-squared (*χ*^2^) test or Fisher’s exact test were used, while the Student’s *t*-test and Wilcoxon rank-sum test were used to test for differences in continuous parameters between groups.

To determine the difference in prediabetes indicators (TyG index and TyG index categories) between the two groups, multiple linear regression (MLR) and logistic regression analysis models were performed. Univariate MLR was adjusted for factors of groups, gender, age, BMI, smoking status, alcohol consumption status, and number of pesticide types used, respectively. Multivariate MLR was further adjusted for group, gender, age, BMI, smoking status, and alcohol consumption status, which have been reported or tested to be associated with the TyG index in previous studies (24, 25). Logistic regression analyses were also performed and adjusted for the same variables (group, gender, age, BMI, smoking status, and alcohol consumption status) as multivariate MLR. Subgroup analysis was performed using stratified multivariate MLR analyses. Statistical significance was defined as *α* < 0.05 for two-tailed value of *p*s.

## 3. Results

### 3.1. General demographic characteristics

General demographic characteristics are presented in Table 1. The average age, BMI, and drinking habits of the two groups were not significantly different (all *p* > 0.05). Age categories, gender, education level, and smoking habits showed significant differences (all *p* < 0.05). The proportion of men, workers with low education levels, and smokers was higher in the G group compared to the F group. The solar greenhouse workers had a lower working age and used more pesticides compared to traditional field workers. The greenhouse worker group owned an average of 2.08 ± 1.11 solar greenhouses and used an average of 6.62 ± 2.56 types of pesticides, which was significantly higher compared to the F group (*p* < 0.05). The types of pesticides used by the two groups are shown in Supplementary Table S1. The use of eight types of pesticides (acetamiprid, imidacloprid, streptomycin, chlorothalonil, carbendazim, propamocarb hydrochloride, procymidone, and avermectin) was more prevalent in the G group compared to the F group, whereas paraquat and glyphosate pesticides were used less (all *p* < 0.05).

**Table 1:** Characteristics of the greenhouse workers (G group) and field workers (F group).

Characteristics	G group	F group	value of *p*
*n* (%)	721 (76.1)	227 (23.9)	–
Gender *n* (%)			0.004^**,b^
Men	299 (41.5)	70 (30.8)	
Women	422 (58.5)	157 (69.2)	
Age (years old)	47.81 ± 8.98	47.74 ± 9.74	0.924^a^
≤ 45 years old	314 (43.6)	80 (35.2)	0.027^*,b^
> 45 years old	407 (56.4)	147 (64.7)	
BMI (kg/m^2^)	23.72 ± 3.46	24.11 ± 3.24	0.137^a^
< 24.0 kg/m^2^	408 (55.3)	119 (52.4)	0.441^b^
≥ 24.0 kg/m^2^	336 (44.7)	108 (47.6)	
Current smoker, *n* (%)	199 (27.6)	44 (19.1)	0.013^*,b^
Alcohol user, *n* (%)	162 (22.5)	39 (17.2)	0.077^b^
Education *n* (%)			0.001^**,b^
Primary school or less	288 (39.9)	85 (37.4)	
Junior high school	339 (47.0)	89 (39.2)	
High school or above	94 (13.1)	53 (23.4)	
Number of solar greenhouses	2.08 ± 1.11	–	–
Working age (years)	13 (8–18)	24 (16–30)	0.001^**,c^
Number of types of pesticides used	6.62 ± 2.56	3.36 ± 2.04	< 0.001^**,a^
TG (mmol/L)	1.51 ± 1.04	1.41 ± 0.95	0.237^a^
FBS (mmol/L)	4.95 ± 0.57	4.88 ± 0.59	0.095^a^
TyG index	8.53 ± 0.56	8.44 ± 0.59	0.033^*,a^
TyG index category			0.107^b^
Low TyG index group	356 (49.4)	126 (55.5)	
High TyG index group	365 (50.6)	101 (44.5)	

BMI, body mass index. TyG index, triglyceride-glucose index. FBS, fasting blood glucose. TG, triglyceride.

^*^p < 0.05, ^**^p < 0.01.

^a^ *t*-test.

^b^ Chi-squared test.

^c^ Mann–Whitney *U* test.

### 3.2. Triglyceride, glucose, and TyG index

**Figure 1:**
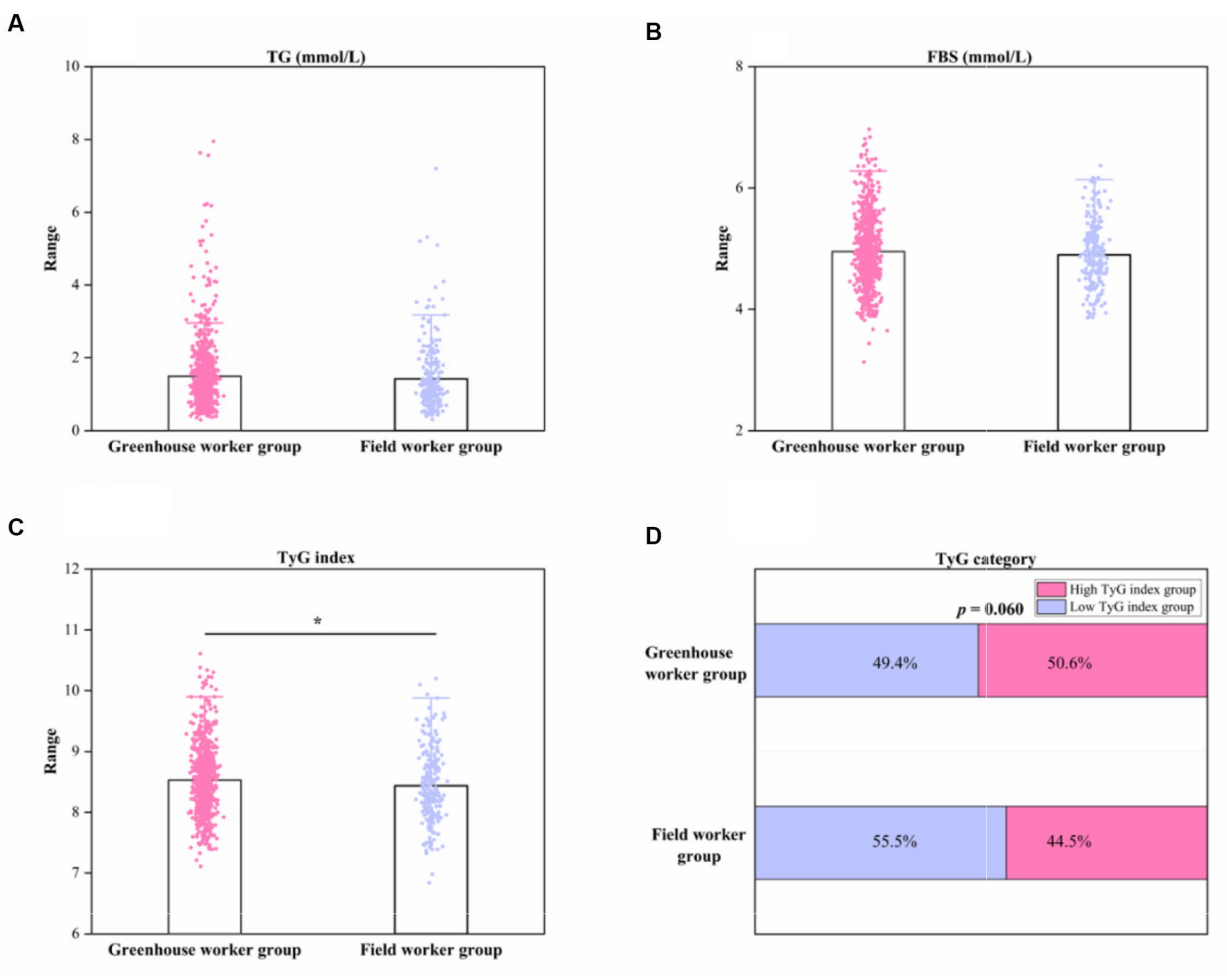
Column diagram and stacked bar chart of TG, FBS, TyG index, and TyG index category between greenhouse worker and field worker groups. **(A)** Column diagram of TG levels between the two groups. **(B)** Column diagram of FBS levels between the two groups. **(C)** Column diagram of TyG index levels between the two groups. **(D)** Stacked bar chart of TyG index category. ^*^p < 0.05. TyG index, triglyceride-glucose index; FBS, fasting blood glucose; TG; triglyceride.

Table 1; Figures 1A,B show that no significant differences in FBS and TG levels were found between the G and F groups (both *p* > 0.05). The mean TG concentration was (1.51 ± 1.04) mmol/L in the greenhouse worker group and (1.41 ± 0.95) mmol/L in the field worker group. The mean FBS concentration was (4.95 ± 0.57) mmol/L in the greenhouse worker group and (4.88 ± 0.59) in the field worker group. However, the TyG index in the G group (8.53 ± 0.56) was higher compared to that in the F group (8.44 ± 0.59) (*p* < 0.05). The proportion of participants with a high TyG index was 50.7% in the G group and 44.5% in the field worker group, but this difference was not significant (*p* > 0.05).

### 3.3. Group differences in the TyG index

**Figure 2:**
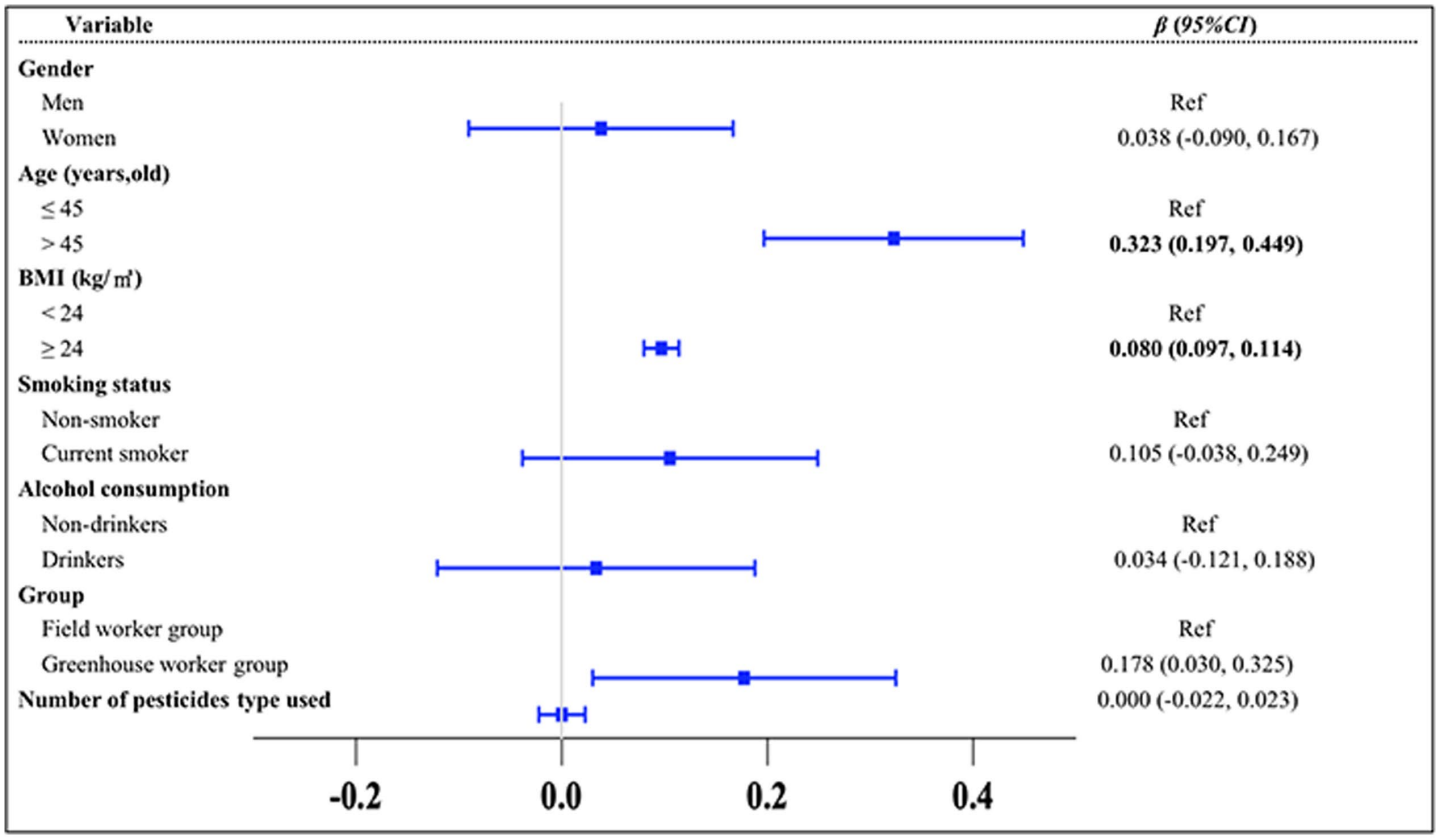
Results of the univariate MLR analysis of the TyG index. Variables of groups (G group and F group), gender, age, BMI, smoking status, and alcohol consumption status were adjusted in the analysis.

**Figure 3:**
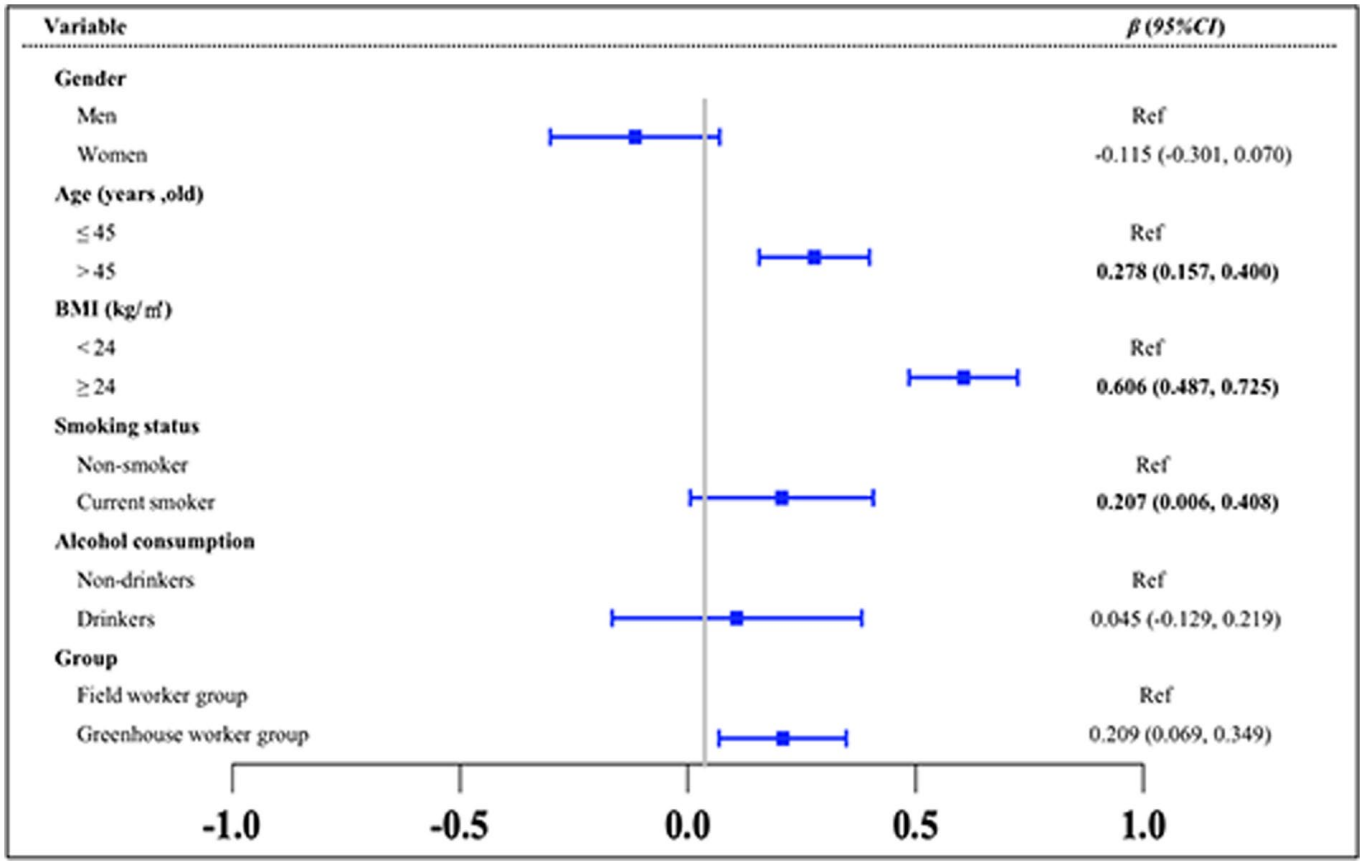
Results of the multivariate MLR analysis of the TyG index. Variables of groups (G group and F group), gender, age, BMI, smoking status, and alcohol consumption status were adjusted in the analysis.

**Figure 4:**
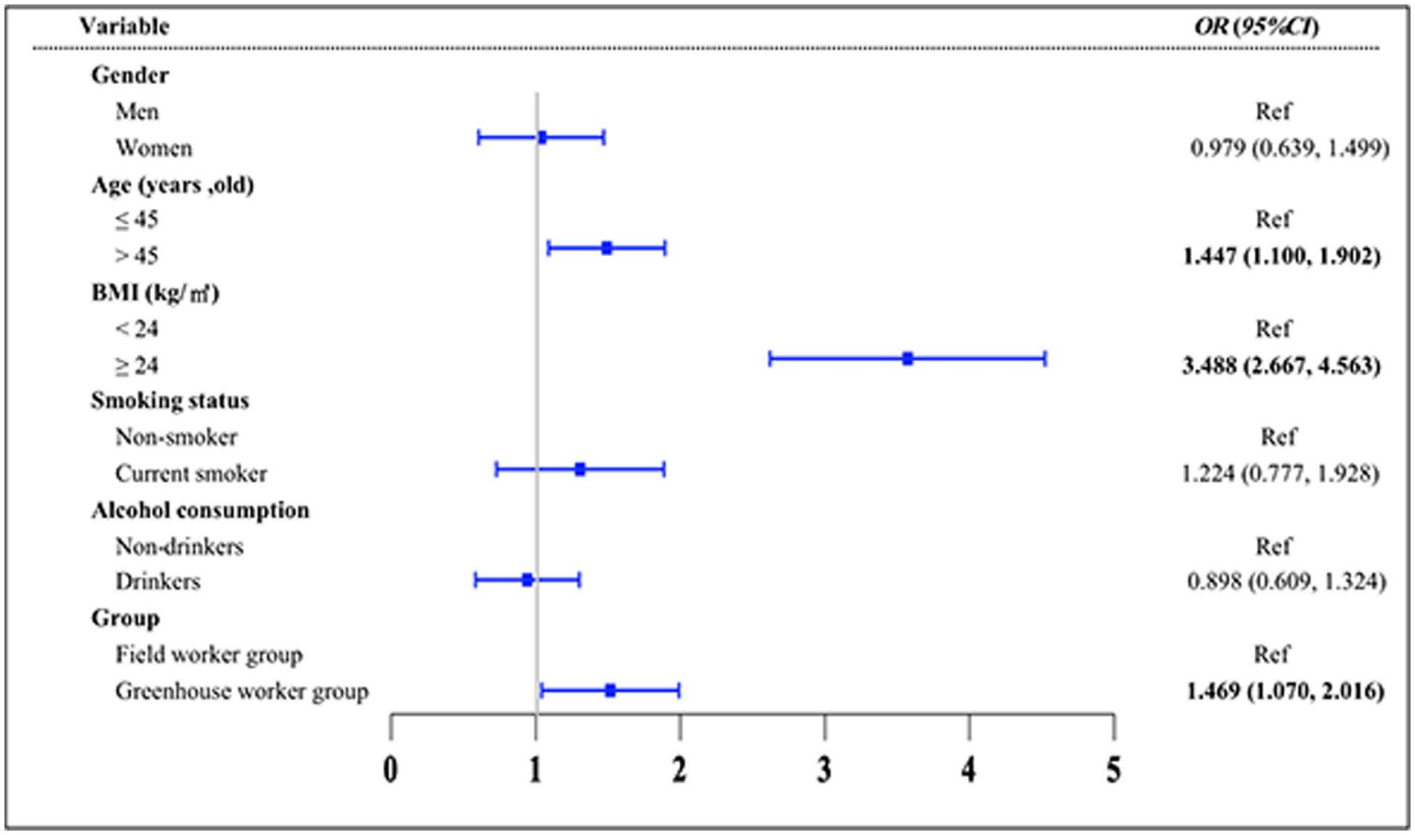
Risk factors for elevated TyG index levels using logistic regression analysis. Variables of groups (G group and F group), gender, age, BMI, smoking status, and alcohol consumption status were adjusted in the analysis.

To explore the differences in the TyG index between the greenhouse workers and field worker groups and to identify key influencing factors, multiple statistical analyses were conducted (Figures 2–4). The results of the MLR showed that the TyG index was positively associated with solar greenhouse work [*β* = 0.178, 95% *CI*: (0.030, 0.325)] compared to field work (Figure 2). Additionally, variables such as age over 45 years and BMI over 24.0 kg/m^2^ were positively associated with an increased TyG index compared to controls [*β* = 0.323, 95% *CI*: (0.197, 0.449), *β* = 0.097, 95% *CI*: (0.080, 0.114), respectively] (Figure 2). However, the associations between the TyG index and 10 different types of pesticides were not statistically significant (all *p* > 0.05) (Supplementary Table S2). After adjustment for confounders reported in the literature (groups, gender, age, BMI, smoking status, and alcohol consumption status), the positive association between TyG index and solar greenhouse work remained robust in the MLR [*β* = 0.209, 95% *CI*: (0.069, 0.349)] (Figure 3).

We categorized the TyG index into two groups based on the median value and found that the workers in the G group had a higher risk of having a TyG index greater than 8.49 compared to the F group [*OR* = 1.469, 95% *CI*: (1.070, 2.016)] (Figure 4). Additionally, age over 45 [*OR* = 1.447, 95% *CI*: (1.100, 1.902)] and a BMI of 24.0 kg/m^2^ or higher [*OR* = 3.488, 95% *CI*: (2.667, 4.563)] were identified as risk factors for a TyG index greater than 8.49 when compared to the control group.

**Figure 5:**
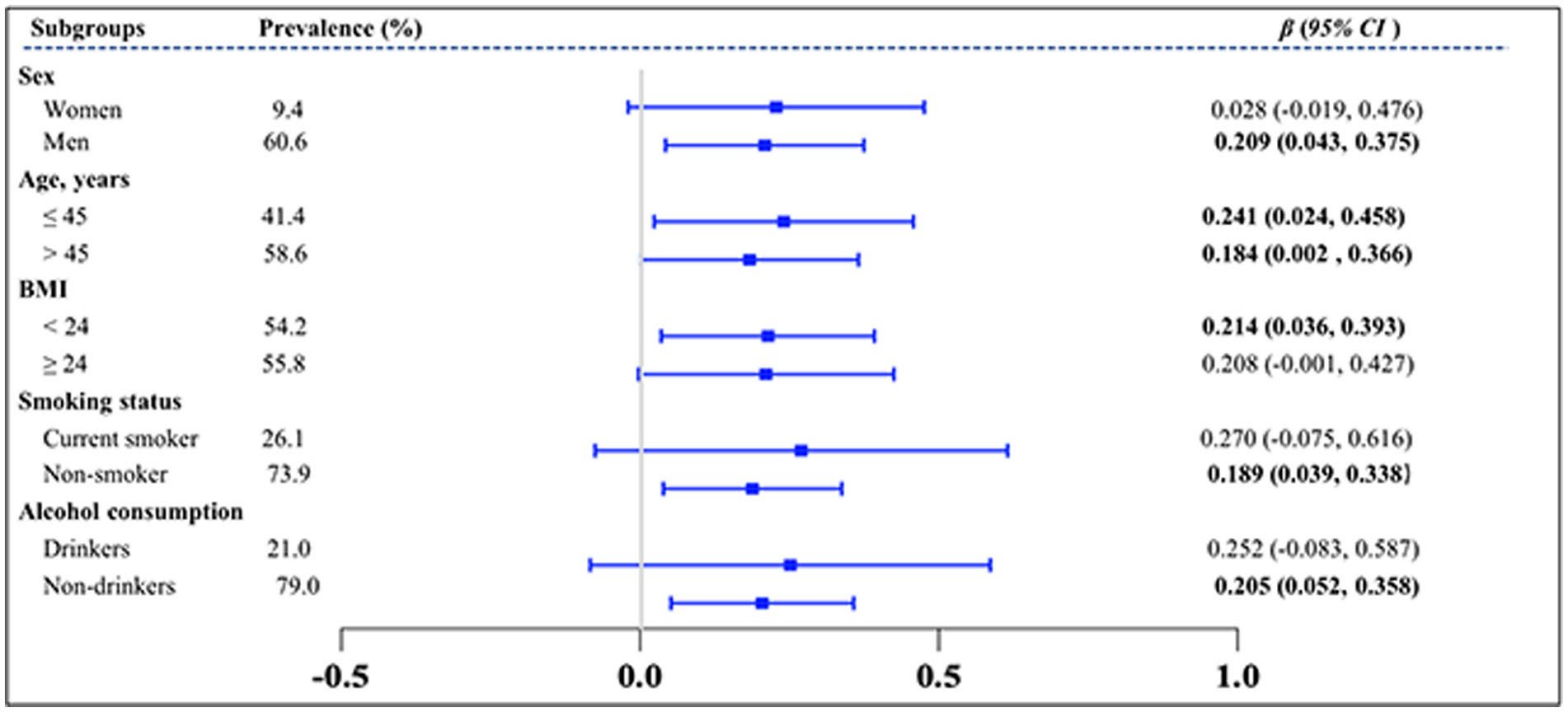
Subgroup analysis of the effect of greenhouse work on TyG index. TyG index, triglyceride-glucose index; VIFs, variance inflation factors. Variables of groups (G group and F group), gender, age, BMI, smoking status, and alcohol consumption status were adjusted in the analysis.

The subgroup analysis further explored the influence of other covariables on the association between the TyG index and solar greenhouse workers and field workers. The results showed a consistent pattern, as illustrated in Figure 5. The TyG index was positively associated with solar greenhouse work, regardless of the subgroups. The effect of the TyG index on the greenhouse worker group was significant among men [women: *β* = 0.228, 95% *CI* (−0.019, 0.476); men: *β* = 0.209, 95% *CI* (0.043, 0.375)], those with a BMI < 24 kg/m^2^ [< 24 kg/m^2^: *β* = 0.214, 95% *CI* (0.024, 0.458); ≥ 24 kg/m^2^: *β* = 0.208, 95% *CI* (−0.001, 0.427)], non-smokers [current smokers: *β* = 0.270, 95% *CI* (−0.075, 0.616); non-smokers: *β* = 0.189, 95% *CI* (0.039, 0.338)], and non-drinkers [drinkers: *β* = 0.252, 95% *CI* (−0.083, 0.587); non-drinkers: *β* = 0.205, 95% *CI* (0.052, 0.358)]. However, the associations of the TyG index with solar greenhouse work were consistent in the two age subgroups [18–45 years old: *β* = 0.241, 95% *CI* (0.024, 0.458); ≥ 45 years old: *β* = 0.184, 95% *CI* (0.002, 0.366)].

## 4. Discussion

In this cross-sectional epidemiologic study, we found that the TyG index was higher in the G group compared to the F group. As far as we know, this is the only study to report the problem of IR and prediabetes among solar greenhouse workers. The current findings suggest that the solar greenhouse work was associated with an increased risk of IR and prediabetes, which should be of guiding significance to exploring occupational worker protection.

There are no reports on the relationship of the solar greenhouse work with IR, prediabetes, diabetes mellitus, or MetS, although studies have been conducted among general farmers, which showed they are probably at increased risk of IR, diabetes mellitus, and MetS compared to the general population (20, 26, 27). Raafat et al. reported that farmers had mean values of insulin resistance (3.19 ± 0.17) higher compared to the general population (1.15 ± 0.04) in Egypt (27), while another study reported that the prevalence of IR (defined as a TyG index above 4.52) was 33.7% in Brazilian farmers (26). The prevalence of diabetes was 9.3% among the rural population in the Republic of Korea, which was higher than that of the general population (8.8%) (28). In this study, we found that the risk of IR was associated with solar greenhouse work.

Evidence suggests that exposure to pesticides is associated with IR in rural populations (19, 26, 27). For example, Bai et al. reported that di-2-ethylhexyl phthalate (a type of OPs metabolite) was positively associated with HOMA-IR (0.18, 0.08–0.28) and IR (HOMA-IR > 2.6) (1.76, 1.17–2.64) (19). Pesticide exposure assessment was not fully performed in this study. The solar greenhouse workers used on average (6.62 ± 2.56) types of pesticides, more than the field workers (3.36 ± 2.04). Exposure to pesticides may be a possible cause of the increased TyG index. OPs exposure induced lipid disorders, an increased TyG index, and free fatty acid synthesis. Dyslipidemia is a key step to IR by activating serine kinases such as protein kinase C and c-jun N-terminal kinase, leading to the inhibition of IRS-1 activity (12, 18). In addition, the environmental temperature in the solar greenhouse is above 25°C, reaching 35°C or more at noon. Heat stress exposure is a potential protective factor for prediabetics. Animal and human studies have shown that heat stress promotes a series of signaling mechanisms (e.g., muscle hypertrophy, angiogenesis, mitochondrial biogenesis, and glucose metabolism) by increasing tissue temperature and enhancing energy turnover (20, 29, 30). While the results showed that the protective effects of environmental heat stress exposure may not be sufficient to offset the impact of an elevated TyG index on solar greenhouse workers, the results of the subgroup analysis consistently demonstrated a positive relationship between the TyG index and solar greenhouse work in all subgroups. These findings suggest that individuals with higher TyG index values are more likely to be engaged in solar greenhouse work, irrespective of subgroup characteristics. Notably, significant associations were observed between the TyG index and solar greenhouse work among specific subgroups. Among men, the effect of the TyG index on the greenhouse worker group was significant, with higher TyG index values associated with an increased likelihood of engaging in solar greenhouse work, which was consistent with the previous report (27).

These findings highlight the importance of the TyG index as a potential indicator for identifying individuals who are more likely to be involved in solar greenhouse work. However, further research is warranted to explore the underlying mechanisms and potential causal relationships between the TyG index and occupational choice in different subgroups. Additionally, considering the cross-sectional nature of this study, future longitudinal studies are needed to establish a temporal relationship between the TyG index and occupational preferences in different populations.

To the best of our knowledge, this is the only study to report that solar greenhouse workers have an increased risk of IR compared with field workers. However, our study still has limitations. First, the information on genetic factors, family history of diabetes mellitus, and hypoglycemic drugs was not adjusted as confounders. Genetic variants involved in the pathogenesis of diabetes mellitus are associated with IR (31), which indicates that it is necessary to explore the associations of solar greenhouse work with IR and genetic predisposition in the future. Second, the exposure assessment of the occupational environment and dietary habits, such as pesticides, temperature, and food types, was not fully conducted. This made it difficult to quantify the contribution of occupational activity and dietary habits in the greenhouse to the increase in the TyG index. Third, it was not possible to make temporal causality inferences between solar greenhouse work and increased risk based on the TyG index in a cross-sectional study.

This study reported on the association of IR with solar greenhouse work. Furthermore, the risk of IR was higher in the workers who were obese and 45 years of age and older than in the controls. These findings suggest that the group of solar greenhouse workers has a higher risk of prediabetes compared to the general farmer population. Occupational hygiene practices programs should be implemented to reduce the risk of prediabetes among solar greenhouse workers.

## Data availability statement

The raw data supporting the conclusions of this article will be made available by the authors, without undue reservation.

## Author contributions

TY: Conceptualization, Data curation, Formal analysis, Funding acquisition, Software, Visualization, Writing – original draft. MW: Writing – original draft. SY: Data curation, Writing – original draft. YW: Data curation, Writing – review & editing. XFZ: Data curation, Resources, Writing – review & editing. XJZ: Conceptualization, Supervision, Writing – review & editing. WM: Investigation, Methodology, Writing – review & editing. ST: Supervision, Writing – review & editing. JL: Supervision, Writing – review & editing.

## Funding

The author(s) declare financial support was received for the research, authorship, and/or publication of this article.

The study was funded by the Beijing Natural Science Foundation, China (7234402) and the Reform and Development Project of the Beijing Academy of Science and Technology (BJAST-RD-BMILP202103&08).

## Conflict of interest

The authors declare that the research was conducted in the absence of any commercial or financial relationship that could be constructed as a potential conflict of interest.

## Publisher’s note

All claims expressed in this article are solely those of the authors and do not necessarily represent those of their affiliated organizations, or those of the publisher, the editors and the reviewers. Any product that may be evaluated in this article, or claim that may be made by its manufacturer, is not guaranteed or endorsed by the publisher.
