# Supplementary material for: Prevalence of insulin resistance in Chinese solar greenhouse and field workers: evidence from a solar greenhouse and field workers study
Source: Front Public Health. 2023 Aug 25;11:1257183. doi: 10.3389/fpubh.2023.1257183 (PMC10485250; doi:10.3389/fpubh.2023.1257183)
Supplement: Supplementary file 1 [file Table_1.DOCX]

**Table S1**. The usage of pesticides in greenhouse worker and field worker groups. *n* (%)

| **Pesticide** | **Greenhouse worker group** | **Field worker group** | ***p-*value** |
| --- | --- | --- | --- |
| *n* (%) | 721 (76.1) | 227 (23.9) | — |
| Imidacloprid | 612 (84.9) | 48 (21.1) | <0.001 ^*^ |
| Acetamiprid | 539 (74.8) | 147 (64.8) | <0.001 ^*^ |
| Chlorothalonil | 648 (89.9) | 27 (11.9) | <0.001 ^*^ |
| Carbendazim | 662 (91.8) | 140 (61.7) | <0.001 ^*^ |
| Procymidone | 504 (69.9) | 6 (2.6) | <0.001 ^*^ |
| Propamocarb hydrochloride | 537 (74.5) | 4 (1.8) | <0.001 ^*^ |
| Streptomycin | 505 (70.0) | 15 (6.6) | <0.001 ^*^ |
| Avermectin | 666 (92.4) | 51 (22.5) | <0.001 ^*^ |
| Paraquat | 217 (30.1) | 166 (73.1) | <0.001 ^*^ |
| Glyphosate | 97 (13.5) | 121 (53.3) | <0.001 ^*^ |

[Note]^*^, *p* < 0.05.
