# Supplementary material for: Prevalence of insulin resistance in Chinese solar greenhouse and field workers: evidence from a solar greenhouse and field workers study
Source: Front Public Health. 2023 Aug 25;11:1257183. doi: 10.3389/fpubh.2023.1257183 (PMC10485250; doi:10.3389/fpubh.2023.1257183)
Supplement: Supplementary file 2 [file Table_2.DOCX]

**Table S2.** Results of multivariate multiple linear regression analysis of TyG index.

| **Pesticides** | ***β*** | ***β* 95% *CI*** | ***p-*value** |
| --- | --- | --- | --- |
| Imidacloprid | 0.033 | -0.068, 0.215 | 0.308 |
| Acetamiprid | -0.015 | -0.179, 0.112 | 0.653 |
| Chlorothalonil | 0.019 | -0.101, 0.187 | 0.557 |
| Carbendazim | -0.007 | -0.201, 0.160 | 0.823 |
| Procymidone | 0.042 | -0.045, 0.217 | 0.197 |
| Propamocarb hydrochloride | -0.021 | -0.175, 0.088 | 0.517 |
| Streptomycin | -0.010 | -0.151, 0.111 | 0.769 |
| Avermectin | -0.004 | -0.162, 0.142 | 0.897 |
| Paraquat | -0.054 | -0.245, 0.021 | 0.098 |
| Glyphosate | -0.036 | -0.242, 0.067 | 0.267 |

Note: ^*^ *p* < 0.05, ^**^ *p* < 0.01.
